# Supplementary material for: A Comparison among Nonvitamin K Antagonist Oral Anticoagulants in Asian Patients with Venous Thromboembolism: A Multi-Institutional Study
Source: J Clin Med. 2022 Dec 1;11(23):7159. doi: 10.3390/jcm11237159 (PMC9738989; doi:10.3390/jcm11237159)
Supplement: Supplementary file 1 [file jcm-11-07159-s001.zip › jcm-1980898-supplementary.pdf]

Table S1. Inclusion criteria defining active cancer in the study cohort

Cancer diagnosis within 6 months before index date
Metastatic cancer
Hematology cancer
Patients treated with radiotherapy or systemic therapy
Patients with consecutive oncology outpatient visits more than twice within one year

Table S2. Codes used to define the cancer population and safety outcomes in the study cohort

	International Classification of Disease 9th revision (ICD-9) code	International Classification of Disease 10th revision (ICD-10) code	Diagnostic Definition
**Cancer**	140-209	C00-C80, C7A, C7B, C81-C96	
**Deep vein thrombosis**	453.4, 453.4x,<br>453.5, 453.5x,<br>453.72, 453.73, 453.74, 453.75<br>453.82, 453.83, 453.84, 453.85	I80.1x, I80.2x, I80.3, I80.8, I80.9<br>I82.4xx, I82.5xx, I82.60x, I82.62x,<br>I82.70x, I82.72x, I82.Axx, I82.Bxx,	
**Pulmonary embolism**	415.1, 415.1x, 416.2	I26, I27.82	
**Intracranial hemorrhage**	430, 431, 432, 852, 853	I60, I61, I62	Discharge
**Gastrointestinal bleeding**	456.0, 456.2, 455.2, 455.5, 455.8, 530.7, 530.82, 531.0–531.6, 532.0–532.6, 533.0–533.6, 534.0–534.6, 535.0–535.6 537.83, 562.02, 562.03, 562.12 562.13 568.81, 569.3, 569.85, 578.0, 578.1, 578.9	K250, K260, K270, K280, K290	Discharge
**Other critical site bleeding**	423,0, 459.0, 568.81, 593.81, 599.7, 623.8, 626.3, 626.6, 719.1, 784.7, 784.8, 786.3	D62, J942, H113, H356, H431, N02, N95, R04, R31, R58	Discharge

Table S3. Baseline demographics and clinical characteristics of patients with VTE treated with DOACs after propensity score weighting

	Apixaban, No. (%) (n = 223)	Dabigatran, No. (%) (n = 48)	Edoxaban, No. (%) (n = 70)	Rivaroxaban, No. (%) (n = 989)	SMD
**Sex**					
Female	126 (56.41)	24 (49.18)	38 (53.94)	561 (56.77)	0.15
Male	97 (43.59)	24 (50.82)	32 (46.06)	428 (43.23)	0.15
**Body weight (kg)**	64.64 ± 14.81	65.79 ± 17.73	62.74 ± 8.59	64.12 ± 14.99	0.22
**BMI**	25.68 ± 8.64	25.96 ± 6.18	24.85 ± 3.77	26.01 ± 16.97	0.22
**Age at index date**					
mean (SD)	67.7 ± 14.64	65.08 ± 17.03	68.44 ± 10.80	67.58 ± 15.38	0.24
18-49	38 (16.94)	11 (22.02)	10 (14.87)	144 (14.60)	0.19
50-64	53 (23.65)	9 (19.73)	15 (20.93)	236 (23.86)	0.100
65-74	46 (20.68)	11 (23.30)	16 (22.48)	235 (23.81)	0.08
75-85	58 (26.19)	14 (29.46)	20 (29.20)	263 (26.58)	0.07
85+	28 (12.55)	3 (5.49)	9 (12.51)	110 (11.15)	0.25
**Type of venous thromboembolism**					
Pulmonary embolism only	88 (39.48)	22 (44.99)	27 (38.09)	342 (34.57)	0.21
Deep-vein thrombosis only	101 (45.23)	21 (43.62)	31 (44.80)	493 (49.86)	0.13
PE and DVT	34 (15.29)	5 (11.38)	12 (17.11)	154 (15.57)	0.16
**Creatinine** (mg/dl), mean (SD)	1.01 ± 0.91	0.86 ± 0.48	0.88 ± 0.26	0.92 ± 0.61	0.21
**eGFR**					
≤30 ml/min	11 (5.01)	2 (3.96)	1 (1.40)	37 (3.71)	0.21
>30 to ≤50 ml/min	48 (21.5)	8 (17.15)	15 (21.66)	218 (22.01)	0.12
>50 ml/min	164 (73.49)	38 (78.89)	54 (76.94)	735 (74.28)	0.13
**Platelet** (1000/μl), mean (SD)	240.73 ± 93.25	253.9 ± 105.19	243.37 ± 75.89	224 ± 103.79	0.29
**Platelet count**					
>100,000 per μl	210 (94.32)	47 (98.17)	67 (96.28)	915 (92.54)	0.270
50,000–100,000 per μl	12 (5.44)	1 (1.83)	2 (3.12)	64 (6.49)	0.24
<50,000 per μl	1 (0.24)	0 (0.00)	1 (0.60)	10 (0.97)	0.140
**Hemoglobin** (g/dL), mean (SD)	10.98 ± 1.78	11.31 ± 1.76	11.09 ± 1.48	11.2 ± 2.06	0.19
**Hemoglobin level**					
>10 g/dL	144 (64.77)	37 (78.01)	44 (63.41)	667 (67.43)	0.33
8–10 g/dL	71 (31.99)	10 (19.80)	22 (31.08)	281 (28.44)	0.28
<8 g/dL	7 (3.24)	1 (2.18)	4 (5.51)	41 (4.13)	0.17
**D-dimer**	2902.81 ± 3641.15	2883.44 ± 3558.07	2255.94 ± 2247.11	3011.9 ± 3847.94	0.240

**AST** (U/L)	39.51 ± 59.92	42.00 ± 71.51	30.51 ± 11.08	38.45 ± 49.43	0.23
**ALT** (U/L)	32.37 ± 49.93	31.57 ± 29.91	27.82 ± 16.38	33.31 ± 44.83	0.16
**Charlson comorbidity index**					
0	39 (17.56)	9 (19.29)	9 (13.22)	177 (17.85)	0.17
1-2	62 (27.73)	13 (26.21)	16 (22.45)	288 (29.09)	0.15
3+	122 (54.71)	26 (54.50)	45 (64.33)	525 (53.06)	0.230
**Comorbidities**					
Active cancer [a]	84 (37.54)	17 (36.34)	36 (50.73)	419 (42.33)	0.29
Myocardial infarction	6 (2.60)	2 (3.98)	1 (0.96)	27 (2.73)	0.2
Stroke	36 (16.03)	8 (15.76)	7 (9.56)	143 (14.49)	0.2
Coronary artery disease	34 (15.39)	7 (14.02)	9 (13.31)	128 (12.97)	0.07
Diabetes mellitus	62 (27.93)	12 (25.48)	20 (29.26)	250 (25.31)	0.09
Hypertension	132 (59.08)	24 (50.79)	37 (53.45)	519 (52.45)	0.17
Liver cirrhosis	10 (4.28)	1 (1.79)	3 (4.95)	31 (3.09)	0.18
Chronic obstructive lung disease	37 (16.76)	6 (11.71)	13 (18.08)	157 (15.88)	0.180
Atrial fibrillation	20 (8.81)	5 (9.69)	6 (8.89)	65 (6.53)	0.12
**Doses of DOACs**					
Standard-dose	111 (49.66)	12 (24.14)	29 (41.65)	528 (53.43)	0.630
Low-dose	112 (50.34)	36 (75.86)	41 (58.35)	461 (46.57)	0.630
**Drug prescribed within 30 days of index date**					
Aspirin	26 (11.53)	4 (8.79)	3 (4.25)	93 (9.40)	0.27
P2Y12 inhibitor [b]	15 (6.72)	3 (6.30)	3 (4.29)	49 (4.91)	0.11
NSAIDs	41 (18.39)	10 (20.01)	13 (18.99)	213 (21.56)	0.08

ALT, Alanine aminotransferase; AST, Aspartate Transaminase; BMI, body mass index; DOACs, direct oral anticoagulants; DVT, deep vein thrombosis; eGFR, estimated glomerular filtration rate; NSAIDs, Non-Steroidal Anti-Inflammatory Drugs; PE, pulmonary embolism; SMD, Standardized Mean Difference; VTE, venous thromboembolism.

[a] Cancer diagnosis within 6 months of the index date, metastatic cancer, hematology cancer, patients with cancer treated with radiotherapy or systemic therapy, patients with recurrent cancer, or patients with more than two oncology outpatient visits within 1 year.

[b] clopidogrel or ticagrelor.

Table S4. Efficacy and safety clinical outcomes after IPTW

	VTE treated with DOAC [a]					HR or SHR for apixaban (95% CI) [b]	HR or SHR for dabigatran (95% CI) [b]	HR or SHR for edoxaban (95% CI) [b]
	Total (n=1330)	Apixaban (n=223)	Dabigatran (n=48)	Edoxaban (n=70)	Rivaroxaban (n=989)			
**Composite effectiveness and safety outcome Recurrent VTE or major bleeding**	143 (10.74)	23 (10.43)	5 (9.57)	6 (8.60)	109 (11.01)	0.944 (0.604-1.477)	0.854 (0.339-2.150)	0.766 (0.340-1.726)
**Effectiveness outcome**								
Recurrent VTE	50 (3.77)	11 (4.98)	3 (5.53)	2 (2.88)	34 (3.47)	1.446 (0.736-2.843)	1.594 (0.464-5.481)	0.828 (0.200-3.426)
Recurrent DVT	28 (2.08)	7 (3.23)	0 (0.00)	1 (1.25)	20 (1.98)	1.636 (0.698-3.834)	NA	0.629 (0.074-5.385)
Recurrent PE	23 (1.75)	4 (1.75)	3 (5.53)	1 (1.63)	15 (1.58)	1.113 (0.367-3.377)	3.533 (0.977-12.776)	1.031 (0.155-6.856)
**Safety outcome**								
Major bleeding [c]	95 (7.11)	12 (5.45)	2 (4.04)	4 (5.73)	77 (7.73)	0.699 (0.381-1.280)	0.515 (0.124-2.139)	0.727 (0.269-1.964)
Intra-cranial hemorrhage	25 (1.90)	6 (2.71)	0 (0.00)	0 (0.00)	19 (1.94)	1.398 (0.561-3.481)	NA	NA
Major GI bleeding	17 (1.25)	0 (0.00)	1 (1.91)	0 (0.00)	16 (1.59)	NA	1.198 (0.148-9.694)	NA
Other critical site bleedings	49 (3.65)	5 (2.27)	1 (2.14)	4 (5.45)	39 (3.91)	0.576 (0.228-1.454)	0.545 (0.076-3.908)	1.395 (0.492-3.959)
Decrease in Hb of ≥ 2 g/dL	8 (0.61)	2 (0.88)	0 (0.00)	1 (0.28)	5 (0.61)	1.460 (0.294-7.264)	NA	0.458 (0.005-41.423)
Death from any cause	173 (12.98)	29 (13.09)	5 (9.80)	7 (10.28)	132 (13.30)	0.979 (0.656-1.462)	0.736 (0.294-1.842)	0.769 (0.363-1.626)

DOAC, direct oral anticoagulants; DVT, deep vein thrombosis; HR, hazard ratio; NA, not applicable; PE, pulmonary embolism; SHR, sub-distribution hazard ratio; VTE, venous thromboembolism.

a Values are presented as n (%).

b With adjustments for all variables listed in eTable 1.

c Major bleeding included major gastrointestinal bleeding, intracranial hemorrhage, bleeding at other critical sites, and a hemoglobin decrease of 2 g/dL or more over 24 hours. For patients who had more than one event, only the first event was counted.

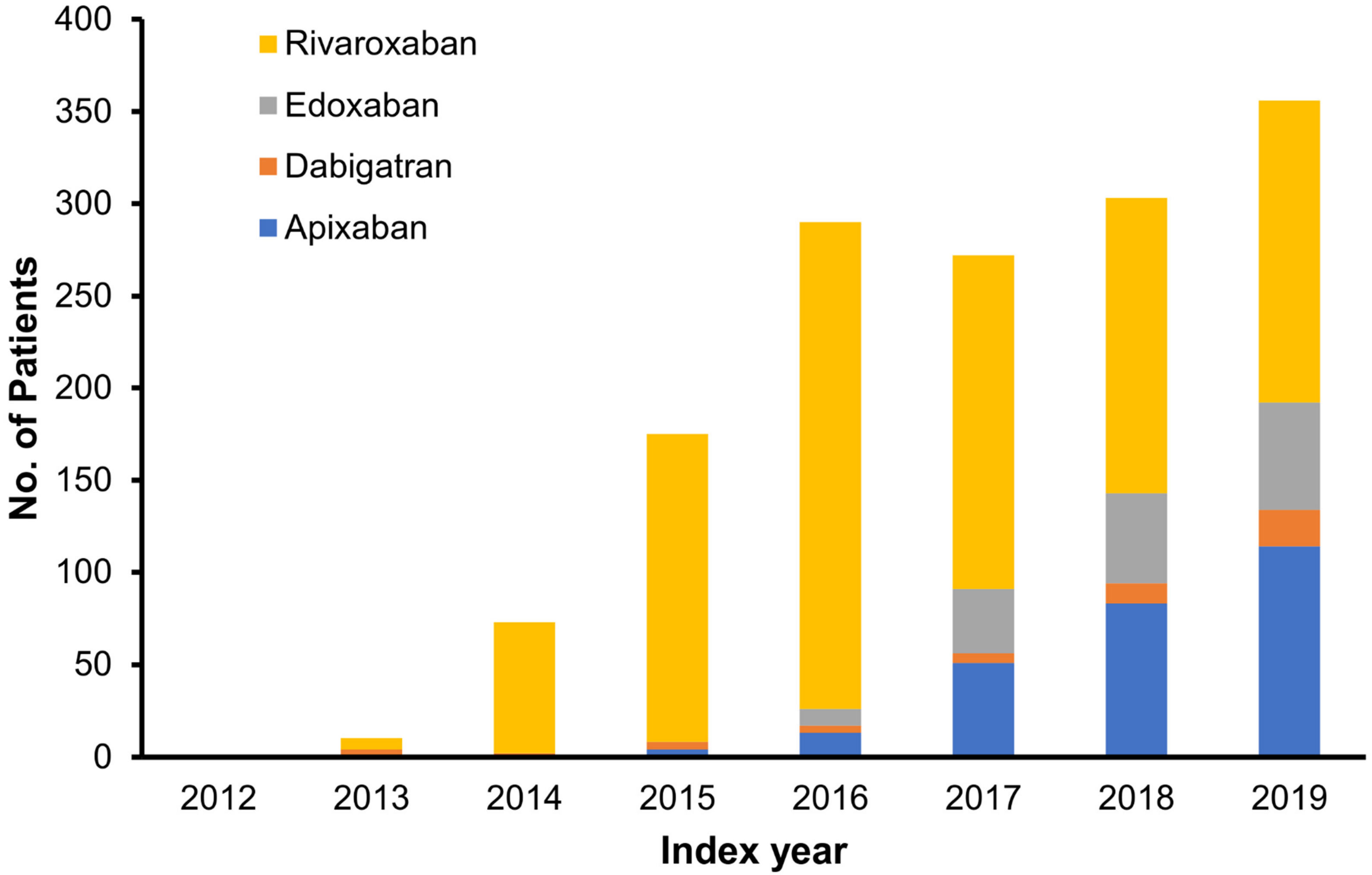

Figure S1. Distribution of DOACs at different time periods in patients with VTE. After 2016, the rate of rivaroxaban use decreased gradually whereas that of edoxaban, dabigatran, and apixaban increased progressively.
